# RON2, a novel gene in *Babesia bigemina,* contains conserved, immunodominant B-cell epitopes that induce antibodies that block merozoite invasion

**DOI:** 10.1017/S0031182019001161

**Published:** 2019-09-13

**Authors:** Juan Mosqueda, Mario Hidalgo-Ruiz, Diana Alexandra Calvo-Olvera, Diego Josimar Hernandez-Silva, Massaro Wilson Ueti, Miguel Angel Mercado-Uriostegui, Angelina Rodriguez, Juan Alberto Ramos-Aragon, Ruben Hernandez-Ortiz, Shin-ichiro Kawazu, Ikuo Igarashi

**Affiliations:** 1 Immunology and Vaccines Laboratory. Facultad de Ciencias Naturales, Universidad Autónoma de Querétaro, Querétaro, Qro, Mexico; 2 U. S. Department of Agriculture, Animal Disease Research Unit, Agricultural Research Service, Pullman, WA, 99164, USA; 3 Facultad de Ciencias Naturales, Universidad Autónoma de Querétaro, Querétaro, Qro, Mexico; 4 CENID-Parasitologia-INIFAP, Morelos, Mexico; 5 National Research Center for Protozoan Diseases, Obihiro University of Agriculture and Veterinary Medicine, Inada, Obihiro, Japan

**Keywords:** *Babesia bigemina*, bovine babesiosis, neutralizing antibodies, peptides, RON2

## Abstract

Bovine babesiosis is the most important protozoan disease transmitted by ticks. In *Plasmodium falciparum*, another Apicomplexa protozoan, the interaction of rhoptry neck protein 2 (RON2) with apical membrane antigen-1 (AMA-1) has been described to have a key role in the invasion process. To date, RON2 has not been described in *Babesia bigemina*, the causal agent of bovine babesiosis in the Americas. In this work, we found a *ron2* gene in the *B. bigemina* genome. RON2 encodes a protein that is 1351 amino acids long, has an identity of 64% (98% coverage) with RON2 of *B. bovis* and contains the CLAG domain, a conserved domain in Apicomplexa. *B. bigemina ron2* is a single copy gene and it is transcribed and expressed in blood stages as determined by RT-PCR, Western blot, and confocal microscopy. Serum samples from *B. bigemina*-infected bovines were screened for the presence of RON2-specific antibodies, showing the recognition of conserved B-cell epitopes. Importantly, *in vitro* neutralization assays showed an inhibitory effect of RON2-specific antibodies on the red blood cell invasion by *B. bigemina*. Therefore, RON2 is a novel antigen in *B. bigemina* and contains conserved B-cell epitopes, which induce antibodies that inhibit merozoite invasion.

## Introduction

Bovine babesiosis is the most important protozoan disease transmitted by ticks. It is caused by intraerythrocytic parasites of the genus *Babesia* that belong to the phylum Apicomplexa. This phylum also includes numerous other pathogens of veterinary and medical importance, for example, *Plasmodium* spp., *Eimeria* spp., and *Toxoplasma gondii.* Apicomplexans are characterized by the presence of apical organelles loaded with molecules that facilitate invasion or escape from host cells (Bock *et al*., 2004; Schnittger *et al*., 2012; Yabsley and Shock, 2013). *Babesia* sporozoites directly invade bovine red blood cells (RBCs), and by binary fission, each develops into two merozoites, which eventually escape from the RBCs into the bloodstream. Each merozoite infects a new RBC to continue the replication cycle (Potgieter and Els, 1977, 1979; Gohil *et al*., 2013). The invasion process consists of four steps: (1) parasite attachment to an RBC; (2) merozoite reorientation, which brings the apical organelles close to the attachment interface; (3) RBC membrane penetration, involving various molecular interactions of the protozoan ligands with the target receptors of the host cell surface; and (4) merozoite internalization. The process is completed when the parasite is inside the RBC (Dubremetz *et al*., 1998; Soldati *et al*., 2001; Yokoyama *et al*., 2006). In each step of the invasion process, *Babesia* parasites secrete proteins from the apical organelles (rhoptries, micronemes, and spherical bodies) towards the invagination site to form moving junctions to the RBC membrane (Yokoyama *et al*., 2006). To date, there are few proteins characterized in *Babesia* species involved in this step of the process. In *Plasmodium falciparum*, AMA-1 is translocated onto the merozoite surface where it can interact with the rhoptry neck protein 2 (RON2), forming a structure known as a ‘moving junction’ (MJ), an irreversible step that commits the parasite to invasion. It is postulated that formation of the MJ is initiated when RON2 is secreted from the rhoptries in a complex formed of RON4, 5, and 8 (Alexander *et al*., 2005; Straub *et al*., 2009; Besteiro *et al*., 2011). This complex is discharged towards the RBC, and RON2 is integrated into the RBC membrane where it acts as an AMA-1 ligand on the parasite surface (Silvie *et al*., 2004; Shen and Sibley, 2012). Blocking this interaction halts merozoite invasion, suggesting that RON2 may be a target for vaccine development (Srinivasan *et al*., 2013, 2014; Zhang *et al*., 2015; Bittencourt *et al*., 2018; Salgado-Mejias *et al*., 2019). Although RON2 has been described in *Babesia divergens, B. microti* and *B. bovis* (Ord *et al*., 2016; Hidalgo-Ruiz *et al*., 2018), to date, there is no evidence of RON2 in other species of *Babesia*, such as *B. bigemina,* where the presence of AMA-1 has been reported (Torina *et al*., 2010). Therefore, the aims of the present study were (a) to identify a homologue of RON2 in *B. bigemina*, (b) to evaluate whether RON2 is transcribed and expressed in merozoites, (c) to determine whether bovines from endemic areas generate antibodies that recognize RON2 conserved epitopes, and (d) to determine the neutralizing activity of specific antibodies.

## Materials and methods

### Identification of the *ron2* gene in the *Babesia bigemina* genome

The *Plasmodium falciparum* RON2 amino acid (aa) sequence (BAH22615.1) was used as a query in a BLASTP search in the BLAST database of the Sanger Institute against the *Babesia bigemina* reference genome (https://www.sanger.ac.uk/resources/downloads/protozoa/babesia-bigemina.html) (Altschul *et al*., 1990). The sequence obtained was analyzed with bioinformatics programs with the following purposes: (a) Identify open reading frames using the ORF finder program (Rombel *et al*., 2002), (b) Determine the signal peptide with the programs SignalP 4.0 (Petersen *et al*., 2011) and SMART (Schultz *et al*., 1998), (c) Find functional domains and their localization with SMART (Schultz *et al*., 1998), (d) Assess whether the predicted protein has transmembrane helices with TMHMM (Krogh *et al*., 2001), and (e) Determine the isoelectrical point and the molecular weight using the CLC Genomics Workbench 7.5 program.

To sequence the full gene, five pairs of primers were designed to amplify overlapping fragments of *B. bigemina ron2* in Oligoanalyzer 3.1 (Owczarzy *et al*., 2008) using the sequence in the Sanger database as a template (Table 1). The combinations used were Fw0RON2-Rv0RON2, which amplified a 913 bp fragment; Fw1RON2-Rv1RON2, which amplified a 701 bp fragment; Fw2RON2-Rv2RON2, which amplified 1,007 bp; Fw3RON2-Rv3RON2, which amplified a 1,045 bp fragment; and Fw4RON2-Rv4RON2, which amplified 627 bp.

**Table 1.** Primers designed for the amplification of Babesia bigemina ron2

Primers	Sequence: 5′ – 3′	Position bp	Amp.
PCR			
Fw0RON2	CACCATGAGAGGATGCGTGC	0 to 16	913 bp
Rv0RON2	GTGTATGCTTGTCTCCTCCCAAT	891 to 913	
Fw1RON2	GAGGTCAAGGAACAACCGAAG	757 to 777	701 bp
Rv1RON2	CTGGGATCAGAGCACACG	1440 to 1457	
Fw2RON2	CGTGTGCTCTGATCCCAG	1440 to 1457	1,007 bp
Rv2RON2	CCTCGTCTGACCATTCCTTG	2427 to 2446	
Fw3RON2	CAAGGAATGGTCAGACGAGG	2427 to 2446	1,045 bp
Rv3RON2	CCTATCCCACTGAACAACGAAG	3450 to 3471	
Fw4RON2	CTTCGTTGTTCAGTGGGATAGG	3450 to 3471	627 bp
Rv4RON2	GATACAAACAGTTAGAGGCTATGG	4053 to 4076	
RT-PCR			
Fwron2	CTGGTGGAGGAGAAAGC	556 to 572	358 bp
Rvron2	GTGTATGCTTGTCTCCTCCCAAT	891 to 913	

Blood from a splenectomized steer infected with *B. bigemina* Chiapas strain was obtained as described previously (Rodríguez-Hernández *et al*., 2012), and the blood was maintained at −20 °C until used. The DNA was extracted using the illustra blood genomicPrep mini Spin Kit (GE Healthcare, Chicago, Illinois, USA) following the manufacturer's protocol. Prior to sequencing, all amplicons were cloned into the pCR^™^ 4-TOPO^®^ vector using the TOPO^®^ TA Cloning^®^ kit (Invitrogen, Carlsbad, California, USA) and transformed into *E. coli* TOP10 cells following the manufacturer's instructions (Invitrogen). Plasmid DNA was used as a template for Taq FS dye terminator cycle sequencing, which was commercially performed at the Instituto de Biotecnologia, Universidad Nacional Autonoma de Mexico (Cuernavaca, Morelos, Mexico), using an automatic DNA sequencer (model 3130xl, Applied Biosystems, Foster City, California, USA). The *B. bigemina* Chiapas strain consensus sequence for RON2 was obtained from the assembly of three cloned sequences. The full *ron2* gene consensus sequence assembly was performed with the CLC Genomic Workbench 7.5 program, and was used in a BLASTp search. The global identity of this sequence with the sequences that showed a similarity in the BLASTp search was calculated with the Pairwise Sequence Alignment tool EMBOSS Needle.

### Transcription analysis

To evaluate the transcription of *ron2* in blood stages, intraerythrocytic parasites were obtained by inoculating 7 mL of blood infected with the Chiapas strain of *B. bigemina* into a splenectomized steer. Five days after the inoculation, the steer was monitored daily, and when the parasitemia reached 4%, determined by microscopic analysis of blood smears stained with Giemsa, whole blood was collected and used for total RNA extraction with Trizol^®^ Reagent (Invitrogen, Carlsbad, California, USA). The mRNA obtained was reverse-transcribed using the Super Script^™^ II kit (Invitrogen, Carlsbad, California, USA) according to the manufacturer's protocol. The cDNA was obtained with an oligo-dT primer and amplified using the following protocol: an initial denaturation at 95 °C for 5 min, followed by 30 cycles consisting of denaturation at 94 °C for 1 min, annealing at 50 °C for 30 s, and extension at 72 °C for 1 min, followed by a final extension at 72 °C for 7 min. The primers Fwron2 and Rvron2 were used, which amplified a 380 bp fragment (Table 1). The amplification was visualized by 1.8% agarose gel electrophoresis stained with ethidium bromide. The amplicon obtained was cloned into the pCR^™^ 4-TOPO^®^ vector using the TOPO^®^ TA Cloning^®^ kit (Invitrogen, Carlsbad, California, USA) and transformed into *E. coli* TOP10 cells as described above. Plasmid DNA was sent for commercial sequencing.

### Selection of peptides containing B-cell epitopes and generation of antibodies against *Babesia bigemina RON2*

Based on the predicted amino acid sequence of RON2, two peptides were selected in conserved regions identified among the sequences obtained of *B. bigemina* RON2 (Chiapas strain and the reference sequence) with multiple sequence alignments using Clustal Omega (Sievers *et al*., 2011), excluding the signal peptide (Schultz *et al*., 1998; Petersen *et al*., 2011) and the hydrophobic, transmembrane or intracellular domains (Krogh *et al*., 2001). B-cell epitopes and antigenic regions were identified using the programs ABCpred (Saha and Raghava, 2006), BCEpred (Saha and Raghava, 2004), and antibody epitope prediction using IEDB (Zhang *et al*., 2008). Two conserved peptide sequences with the highest value in all three algorithms were selected as peptides: Peptide A (IPSVNPLYTRMTPDERKVEFQQ) and Peptide B (FGRVVPPPVYNNKWKR). Both peptides were commercially synthetized as a multiple antigen peptide system of 8 branches (MAPS-8) by GL Biochem (Shanghai, China). To produce antisera against each individual peptide, two New Zealand male rabbits were immunized with each peptide. Four immunizations were applied and each dose was inoculated subcutaneously near the iliac lymph nodes, with 100 *µ*g of each synthetic peptide suspended in 0.5 mL of PBS at pH 7.4 and emulsified with 0.5 mL of Montanide ISA 50 V2 adjuvant (Seppic, Puteaux, France). The immunizations were performed every 15 days, and serum samples were obtained before each immunization. A final serum sample was obtained 15 days after the last immunization. All serum samples were stored at −20 °C until use. All animal handling and experimentation were performed under the UAQ's Bioethics Committee procedures with the approval number FCN/2011-0221.

### Expression analysis

To evaluate the expression of RON2, a Western blot analysis was performed. For this, a pellet of *B. bigemina*-infected erythrocytes (iRBC) was washed five times in ice-cold PBS containing protease inhibitors (Roche-Applied Science, Penzberg, Upper Bavaria, Germany). Each washing step consisted of keeping the iRBC on ice for 5 min, mixing with vortex every 20 s, and then centrifuging the iRBC at 1940 × ***g*** at 4 °C. The supernatant was discarded, and the pellet was suspended in 500 *µ*L of ice-cold PBS containing protease inhibitors. Freezing and thawing occurred at the end of each washing step. At the end of this procedure, the sample was centrifuged at 7500 × ***g*** at 4 °C for 5 min, the supernatant was discarded, and the pellet was suspended carefully in 50 *µ*L of lysis buffer (50 mm Tris-l, 150 mm NaCl, 0.5% Triton X-100, 10 mm EDTA) and mixed with 100 *µ*L of protein loading buffer. This mix was boiled for 5 min and centrifuged briefly. Using 15 *µ*L of this mix per well, an SDS-PAGE (8%) was performed (100 volts, 3 h). Then, the proteins were transferred to a nitrocellulose membrane for 1 h at 100 volts. The membrane was washed with TBS for 5 min and blocked with TBS with 5% skim milk (TBS-M) for 2 h at room temperature. The rabbit anti-RON2 antiserum was diluted at 1:250 in TBS-M (2%) and incubated with the membrane overnight at 4 °C. The membrane was washed two times with TBS-M (2%) and incubated and blocked again for 1 h. The membrane was incubated with a donkey anti-rabbit IgG antibody conjugated with HRP (Jackson ImmunoResearch, West Grove, Pennsylvania, USA) diluted 1:5000 in TBS-M (2%) for 1 h in agitation at room temperature. The membrane was washed three times with TBS and two times with TBS and 0.1% Tween (TBS-T). All washes were agitated at room temperature. Finally, the reaction was developed with ECL (GE, Boston, MA, USA) in autoradiography (X-ray) films (Santa Cruz, Dallas, Texas, USA). Commercial protein standards were used as reference to estimate the molecular weight (PageRuler Plus, Thermo Scientific, Waltham, Massachusetts, USA). As controls, uninfected bovine erythrocytes were incubated with post-immune serum and *B. bigemina* infected erythrocytes were incubated with pre-immune serum.

A confocal microscopy analysis was performed with each antiserum. For this, the Texas strain of *Babesia bigemina* was maintained *in vitro* with daily changes of complete medium, consisting of M199 medium (Sigma-Aldrich, St. Louis Missouri, USA) supplemented with 40% bovine serum and antibiotic-antimycotic (Sigma-Aldrich, St. Louis Missouri, USA). When the parasitized erythrocytes reached >4%, iRBCs were washed with M199 and resuspended in VYM solution. Smears were made in ProbeOn slides (Fisher Scientific, Ontario, Canada) and fixed with methanol for 5 min. The slides were stored at −80 °C until used. Each slide was dried and fixed with 90% acetone 10% methanol for 1 h at −20 °C. The tissue was blocked with 5% horse serum in PBS – 0.2% Tween-20 (PBS-T). Then, they were incubated with each rabbit anti-RON2 antiserum diluted 1:50 in PBS-T for 1 h at 37 °C, followed by ten washes with PBS-T. A second incubation was performed with a goat anti-rabbit IgG antibody coupled with Alexa-488 (Thermo Scientific, Waltham, Massachusetts, USA) diluted 1:200 in PBS-T containing Hoechst 33 342 for nuclei staining (Thermo Scientific, Waltham, Massachusetts, USA) for 1 h at 37 °C, followed by ten washes with PBS-T. As negative controls, rabbit preimmune sera were used in the same conditions. The slides were mounted with ImmunoSelect antifade mounting medium (Dianova, Hamburg, Germany) and a coverslip. Each slide was analyzed in a confocal microscope (Leica TCS SP5 Confocal Laser Scanning Microscope) using lasers specific for Alexa-488, Hoechst 33 342 and brightfield. Images were processed and merged with the LAS Advanced Fluorescence software (Leica, Wetzlar, Alemania).

### Recognition of RON2 peptides by antibodies from naturally infected bovines

To assess the presence of antibodies to *B. bigemina* RON2 in naturally infected bovines, we analyzed the serum of bovines from endemic areas and positive for *B. bigemina*. First, serum samples collected from bovines living in endemic areas from different locations in four different states of Mexico and positive for *B. bigemina* infection were tested against each RON2 peptide by an indirect ELISA. For this, one hundred and twenty-one bovine serum samples were first analyzed by the indirect immunofluorescence test (IFAT) to confirm exposure: 115 were positive for *B. bigemina* and 6 were negative for the presence of anti-*B. bigemina* antibodies. None of the sera included in this experiment were positive to the presence of antibodies anti*-B. bovis* by IFAT. The protocol for immunofluorescence has been published elsewhere and the cut-off dilution value was 1:80 (OIE – World Organisation for Animal Health, 2019). Additionally, sera from three bovines born and raised in a tick-free area and negative to *B. bigemina* by both IFAT and nested PCR were used as negative controls (Figueroa *et al*., 1993). Each peptide was covalently bound to Pierce^®^ amine-binding, maleic anhydride ELISA plates (Thermo Scientific, Waltham, Massachusetts, USA) according to the manufacturer's protocol. The plates were activated by washing them three times with PBS, pH 7.4. Then, 100 *µ*L of each peptide at 10 *µ*g mL^−1^ in PBS pH 7.4 was added to each well, and the plates were incubated overnight at 4 °C. Each well was blocked with 100 *µ*L of SuperBlock™ blocking buffer (Thermo Scientific, Waltham, Massachusetts, USA) for 60 min at 37 °C. A total of 100 *µ*L of each bovine serum diluted 1:50 was added to each well and incubated for 60 min at 37 °C. The plates were washed three times with PBS-T and incubated with 100 *µ*L of donkey anti-bovine IgG antibody conjugated with alkaline phosphatase (Jackson ImmunoResearch, West Grove, Pennsylvania, USA) diluted 1:500 in PBS, pH 7.4. After an incubation period of 60 min at 37 °C, the plates were washed three times. Each plate always included a blank sample and a negative control serum in the same position. Finally, the reaction was revealed with OPD (Sigma-Aldrich, St. Louis Missouri, USA), and after an incubation period of 20 min at room temperature, the reaction was read at 450 nm with an iMark Microplate Absorbance Reader with the Microplate Manager^®^ 6 Software (Bio-Rad Laboratories, Richmond, California, USA). Each serum sample was analyzed in triplicate, and the cut-off value of the test was determined using the mean OD value of triplicate wells plus 3 standard deviations (s.d.) of the negative control serum samples. All the OD values below this cut-off value were considered negative.

### Neutralization assay

To test the capacity of RON2 antibodies to block merozoite invasion, an *in vitro* neutralization assay was performed. For this, the *Babesia bigemina* Puerto Rico strain was cultured essentially as described by Levy and Ristic (Levy and Ristic, 1980) with modifications as follows: This strain was cultured in 96-well plates using HL-1 medium supplemented with 5% bovine red blood cells, 40% bovine serum, 0.1 M TAPSO, and a pH of 7.2. The cultures were incubated at 37 °C and 5% CO_2_. When the cultured parasites reached 6% parasitized erythrocytes, approximately 1 × 10^6^ iRBCs contained in 16.5 *µ*L were added to fresh medium supplemented with normal red blood cells and serum. Cultures were prepared in triplicate for each neutralization assay, and after inactivating the complement by heating at 56 °C for 30 min, each rabbit antiserum against RON2 was added in a 1:5 proportion to each well. The amount of a normal rabbit serum added to the culture was tested previously to avoid interference with culture development. The statistical analysis demonstrated that there was no significant difference between the control culture without rabbit serum and the culture tested with a 1:5 serum proportion (data not shown). The cultures were incubated at 37 °C in 5% CO_2_ for 48 h, and a drop of homogenized culture was obtained and used to prepare smears, which were fixed in methanol and stained with Giemsa. The percentage of parasitized erythrocytes (PPE) was determined by counting the infected and noninfected red blood cells in five representative fields (Figueroa and Buening, 1991; Hines *et al*., 1992). A student's *t*-test was carried out to make a comparative media analysis of nonpaired samples to test differences between the culture supplemented with preimmunization sera and the postimmunization sera. The data were analyzed using SPSS 22.0 Software.

## Results

### Babesia bigemina has a ron2 gene

**Fig. 1.**
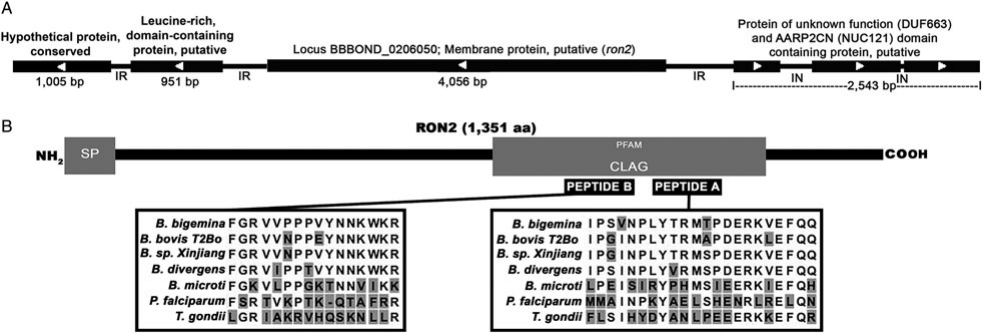
Genome location and bioinformatics analysis of B. bigemina ron2. (A) Position of ron2 in chromosome II. BLASTP analysis identified a sequence in GenBank (CDR95447.1) referred to as the ‘putative membrane protein of B. bigemina” in the locus BBBOND_0206050. (B) Results of the SMART and Pfam analysis of the predicted RON2 protein showing the signal peptide (SP) and the functional CLAG domain (gray boxes). The position of the selected peptides A and B in the domain is indicated with black boxes and the alignment of several apicomplexan species for peptide A and B sequences.

The amino acid sequence of *Plasmodium falciparum* RON2 was used as a BLASTP query in the Sanger Institute database before the genome was annotated and migrated to NCBI (Altschul *et al*., 1990). We found an ORF of 4056 bp in the *Babesia bigemina* genome with 27.82% identity (87% Coverage). The predicted protein contained 1351 aa, a putative signal peptide sequence in the N-terminal region from aa 1 to 26, a CLAG domain comprised of amino acids 718 to 1162 (Fig. 1) and a region of three hydrophobic domains which failed to reach a predicted value for transmembrane helices from amino acids 1093–1112, 1143–1160 and 1214–1232 (not shown). The mature protein had an expected molecular weight of 149 kDa and an isoelectric point of 9.38. Currently, *B. bigemina* RON2 in the NCBI is CDR95447.1. This is a single copy gene located on chromosome II (LK391708.1) (Fig. 1). The percentage of global identity between RON2 of the Chiapas strain (AQU42588.1) with other homologous sequences that showed a similarity in the BLASTp search are shown in Table 2. These results demonstrate the presence of a *ron2* gene in the *B. bigemina* genome. Importantly, the predicted protein sequence contained the typical structure and features of RON2 present in other Apicomplexa parasites.

**Table 2.** Percentage of global identity of B. bigemina RON2 Chiapas strain (AQU42588.1) with other homologues proteins

Organism	Protein	NCBI Accession	Coverage (%)	Identity (%)
*B. bigemina* reference strain	*Membrane protein, putative*	CDR95447.1	100	99.0
*B. ovata*	*Rhoptry neck protein*	XP_028867615.1	100	92.1
*B. orientalis*	*RON2*	AWO67479.1	99	67.5
*B. bovis T2Bo*	*Hypothetical protein*	XP_001608815.1	99	64.1
*B. divergens*	*Rhoptry neck protein 2*	ADM34975.2	99	63.6
*B.* sp. *Xinjiang*	*Rhoptry neck protein 2*	XP_028870902.1	75	57.7
*B. microti strain RI*	*Rhoptry neck protein 2*	XP_021338832.1	75	28.8

### RON2 is transcribed and expressed in *Babesia bigemina*

**Fig. 2.**
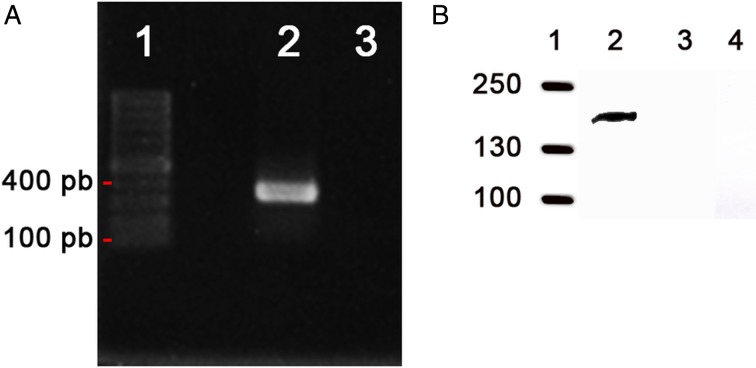
*Babesia bigemina ron2* is transcribed and expressed in erythrocytic stages. Panel A. RT-PCR was visualized on a 1.8% agarose gel stained with ethidium bromide using a pair of primers to amplify a 358 bp fragment. Lane 1: DNA ladder marker; Lane 2: *B. bigemina* mRNA with reverse transcriptase; Lane 3: *B. bigemina* mRNA without reverse transcriptase. Panel B. Western blot showing a specific band of approximately 149 kDa detected by anti-RON2 antiserum. Lane 1. Prestained Protein Ladder shown in kiloDaltons; Lane 2. Total extracts of iRBCs; Line 3. Total extracts of noninfected RBCs. Line 4. Total extracts of iRBCs incubated with pre-immune serum.

There are no reports on the expression of the *ron2* gene in *B. bigemina* to date. To evaluate the expression, the erythrocytic stages of *B. bigemina* were first analyzed for mRNA transcription. As observed in Fig. 2, Panel A, cDNA of *B. bigemina*-infected erythrocytes was amplified by PCR, showing a band of the expected size (380 bp) in agarose gel electrophoresis. The cDNA sequence obtained was 100% identical to the *B. bigemina* RON2 (accession number: KU696964, data not shown). No amplification was observed when the same mRNA sample was amplified without reverse transcriptase, indicating specific amplification of cDNA but not DNA, thus confirming that the *ron2* gene is transcribed in erythrocytic stages of *B. bigemina*. Second, erythrocytic stages were analyzed for protein expression by Western blot. For this, a RON2 antiserum identified a specific band with a molecular weight equivalent to the predicted weight of 149 kDa (Fig. 2 Panel B, lane 2). No signal was observed when the same antiserum was incubated with proteins from uninfected red blood cells, nor when infected erythrocytes where incubated with pre-immune serum, used as control (Fig. 2, Panel B, lanes 3 and 4, respectively). These results confirm that the antibodies generated against RON2 specifically recognize a protein band of the expected molecular weight of RON2 in *B. bigemina*-infected RBCs.

**Fig. 3.**
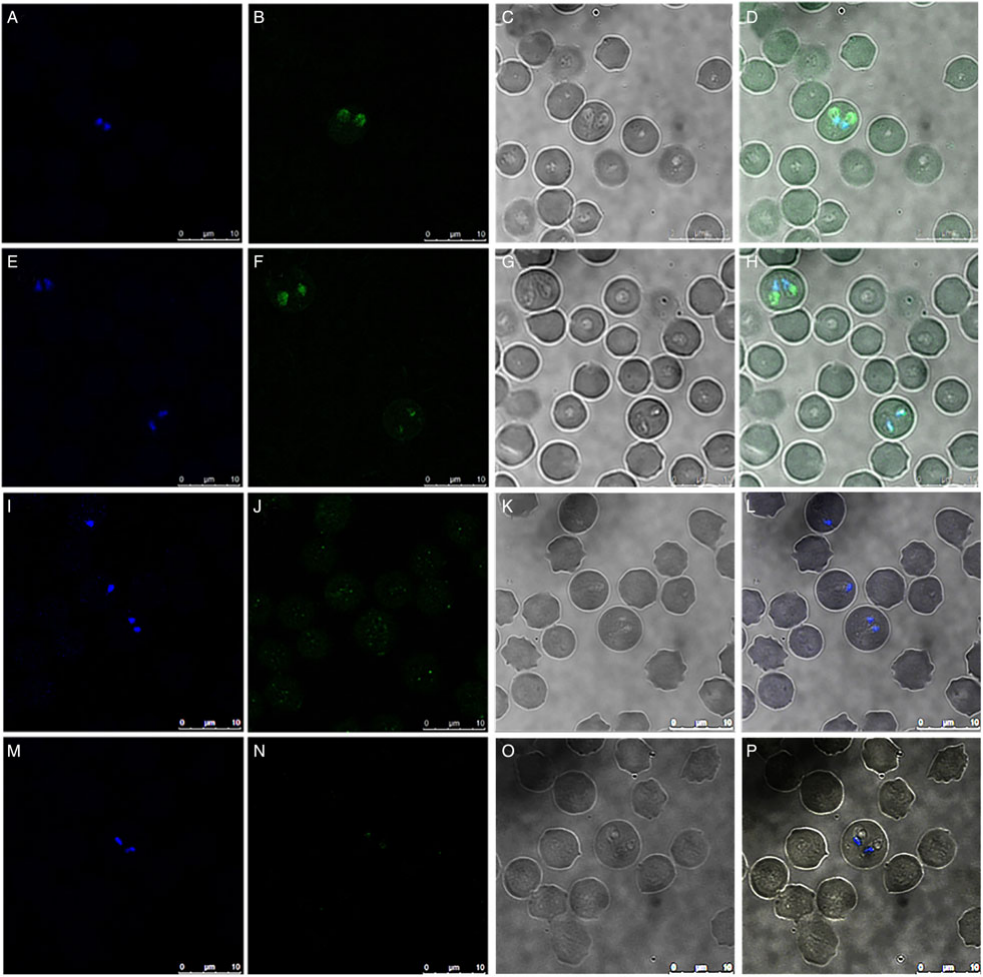
RON2 is expressed in the apical end of B. bigemina merozoites. Intraerythrocytic parasites were incubated with rabbit antiserum against peptide A (Panels B and D) or rabbit antiserum against peptide B (Panels F and H). No signal was observed when merozoites were incubated with the preimmunization serum from each rabbit for peptide A (Panels J and L) or peptide B (Panels N and P). Nuclei were stained with Hoechst 33342 (Panels A, E, I M). Bright field images (Panels C, G, K O) were also used to obtain merged images (Panels D, H, L, P). Bar = 10 *µ*m.

Additionally, anti-RON2 antisera were evaluated by confocal microscopy to determine the expression pattern of RON2 in the merozoite stage. Rabbit antisera generated against two RON2 peptides were used to identify intraerythrocytic merozoites. Using a rabbit anti-serum for each peptide, merozoites were recognized by the respective antiserum (Fig. 3, Panels B and F). In contrast, as expected, no signal was detected when the parasites were incubated with preimmunization sera used as controls (Fig. 3, Panels J and N). A pattern consisting of a defined and intense stain was observed towards the apical end of each paired merozoite, right after the nucleus, where typically, the apical organelles, including the rhoptries, are located (Fig. 3, Panels B, D, F, and H). Together, these results confirm that RON2 is expressed in *B. bigemina* blood stages and that antibodies against RON2, specifically recognize the protein in intraerythrocytic merozoites.

### RON2 has conserved B-cell epitopes that are recognized by naturally infected bovines

RON2 is a highly conserved protein in other Apicomplexa protozoa and is secreted during host cell invasion. To determine whether cattle naturally infected with *B. bigemina* generate antibodies against RON2, two peptides containing conserved, predicted B-cell epitopes were exposed to serum samples from *B. bigemina*-infected bovines obtained from endemic areas. As shown in Table 3, one hundred and fifteen serum samples from naturally infected bovines were analyzed. Our results indicate that 113 out of 115 (98.26%) cattle serum samples contained antibodies against peptide A, and 114 out of 115 (99.13%) serum samples contained specific antibodies against peptide B. These sera samples are from naturally infected cattle from different geographical regions as it is shown in Table 3. Two bovines with antibodies against *B. bigemina* did not recognize peptide A, while one bovine failed to recognize peptide B. These animals were not the same nor from the same farm. The six negative serum samples analyzed did not react with either of the two peptides.

**Table 3.** Presence of antibodies against RON2 peptides in B. bigemina naturally infected bovines

Estate	Total	Farm/Ranch	Peptide A		Peptide B	
			‘+’	‘−’	‘+’	‘−’
Aguascalientes	38	Villa Guadalupe	8	1	9	0
		Rancho las Palomas	24	1	25	0
		Granja María I	4	0	4	0
Querétaro	11	Rancho la Soledad	4	0	3	1
		Granja Araceli	7	0	7	0
Sinaloa	29	El Torito	8	0	8	0
		La Herradura	3	0	3	0
		Rancho el Moral I	3	0	3	0
		Rancho el Moral II	11	0	11	0
		El Barón	4	0	4	0
Veracruz	37	Playa Vicente	2	0	2	0
		El Arbolito	3	0	3	0
		Manuel A. Nielda	2	0	2	0
		La Esperanza	3	0	3	0
		Irineo Murillo	4	0	4	0
		Las Torres	6	0	6	0
		El Orijuelo	3	0	3	0
		San Fandila	12	0	12	0
		Buenos Aires	2	0	2	0
Total	115		113	2	114	1

‘+’, Positive; ‘−’, Negative.

### Neutralization assay

**Fig. 4.**
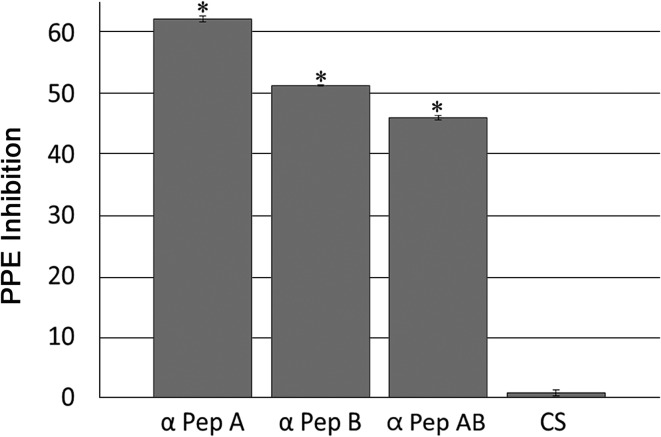
Neutralization assay using antibodies against *B. bigemina* RON2. The percentage of parasitized erythrocytes (PPE) inhibition was determined in *B. bigemina* cultures supplemented with antibodies anti-peptide A (*α* Pep A), antibodies anti-peptide B (*α* Pep B), and a mix of antibodies to both peptides (*α* Pep AB). Serum from a rabbit immunized only with adjuvant was used as a control serum (CS). All data are expressed as percentage of parasitized erythrocytes inhibition considering all the cells counted in five representative fields as the total. The inhibition percentage for each treatment was as follows: peptide A: 62.22%; peptide B: 51.28% and peptide A + B mix: 46.04%. The asterisks indicate the values that are significantly different from the control and cultures with preimmune serum (*P* < 0.05).

To evaluate the capacity of specific antibodies against RON2 to block merozoite invasion, a neutralization assay was carried out. *Babesia bigemina in vitro* cultures containing antibodies against RON2 showed a statistically significant difference in the percentage of inhibition in comparison to that of the culture supplemented with preimmunization serum (Fig. 4). The antibodies against peptide A induced the highest neutralization activity with a 62.22% reduction of PPE (culture with pre-immune serum: 7.65% PPE, culture with post-immunization serum: 2.89% PPE) (*P* < 0.05), while the anti-peptide B antibodies reduced the PPE by 51.28% (culture with pre-immunization serum: 7.16% PPE, culture with post-immunization serum: 3.49% PPE) compared to that of their respective preimmune sera (*P* < 0.05). Furthermore, we analyzed the inhibition capacity of both antisera mixed in a 1:1 proportion, and the results of this assay showed a 46.04% reduction of PPE (culture with pre-immune serum: 4.29% PPE, culture with post-immunization serum: 2.28% PPE) (*P* < 0.05) (Fig. 4). The antiserum of a rabbit immunized with adjuvant alone used as control serum (CS) induced a 0% reduction of PPE (culture with pre-immunization serum: 3.25% PPE, culture with post-immunization serum: 3.3% PPE) compared to the respective preimmunization serum used as control for a possible adjuvant effect. Together, these results show that antibodies against RON2 reduce *B. bigemina* invasion of erythrocytes, suggesting a role for RON2 in the invasion process.

## Discussion

To date, RON2 has not been identified in *B. bigemina*, and the present work represents the first report of the identification, transcription and expression of this protein in *B. bigemina*. The moving junction (MJ) is the irreversible interaction between AMA-1 and RON2 in Apicomplexa parasites, and both proteins have an important role in parasite invasion of erythrocytes (Richard *et al*., 2010; Srinivasan *et al*., 2013; Bermúdez *et al*., 2018; Salgado-Mejias *et al*., 2019). AMA-1 and RON2 were initially characterized in *Toxoplasma* and *Plasmodium* (Curtidor *et al*., 2011); where RON2 is integrated into the RBC membrane and there it is used as an AMA-1 ligand on the cell surface (Silvie *et al*., 2004; Shen and Sibley, 2012). AMA-1 is a protein required for invasion of the host cell (Remarque *et al*., 2008) and has been described previously in *B. bigemina* (Torina *et al*., 2010). In this study, we focused on the identification and characterization of the RON2 protein. The *ron2* gene was identified as a single copy gene, using an initial bioinformatics approach, and the full sequence of the gene was amplified using several sets of primers. The mature predicted protein contains 1351 aa, excluding the signal peptide. RON2 proteins are highly conserved among different species of the phylum Apicomplexa. RON2 proteins in Apicomplexa species share some structural and functional characteristics, such as a signal peptide and a CLAG domain (Kaneko *et al*., 2005; Rungruang *et al*., 2005; Ghoneim *et al*., 2007; Cao *et al*., 2009). *Babesia bigemina* RON2 also contains these features, including the CLAG domain. This domain identified as pfam03805 is part of a gene family in *P. falciparum*, it is found in at least, nine proteins that are expressed in blood stages. Some proteins with this domain have been related to the cytoadherence to endothelial receptors in the sequestration of iRBCs in blood vessels of the brain causing cerebral malaria. Other proteins with this domain have been described as essential for the binding of merozoites to RBCs or in the invasion of midgut lumen cells and salivary gland cells by sporozoites (Holt *et al*., 1999). Interestingly, by bioinformatics, we did not find the three transmembrane domains in *B. bigemina* RON2 as they were found in *B. bovis* (Hidalgo-Ruiz *et al*., 2018). Instead, three hydrophobic domains were predicted from amino acids 1093–1112, 1143–1160 and 1214–1232. These transmembrane domains are used in other species as ligands for AMA-1 (Richard *et al*., 2010; Srinivasan *et al*., 2013; Bermúdez *et al*., 2018; Salgado-Mejias *et al*., 2019), suggesting that, although the function of this protein is also conserved in this species, the topology of *B. bigemina* RON2 could be not the same as that of *B. bovis* RON2. More functional studies are necessary to test this hypothesis.

Due to the implication of RON2 in the erythrocyte invasion process, in this study, we determined whether *B. bigemina ron2* was a functional gene; therefore, we analyzed its transcription and expression in the blood stages of the parasite. Transcripts of *ron2* were detected by RT-PCR in blood stages of *B. bigemina*, and a defined band of the expected molecular weight of the mature protein (149 kDa) was detected in blood stages as well by Western blot. Therefore, we conclude that RON2 is a functional gene and is expressed in the erythrocytic stages of *B. bigemina*. Additionally, specific antibodies against two conserved RON2 peptides were generated and evaluated on native antigen by confocal microscopy. We successfully generated antibodies against RON2, which bound to intraerythrocytic merozoites. The expression pattern observed consisted of an intense localization in the anterior end of paired merozoites, with no staining in the posterior end and this staining pattern was consistent with that observed for *B. divergens* (Ord *et al*., 2016). In other Apicomplexa merozoites, this protein is stored in the anterior rhoptry neck (Proellocks *et al*., 2010), which might explain the localization in the apical end. More specific experiments, including electron microscopy are necessary to identify the exact subcellular localization of RON2 in *B. bigemina* merozoites. The results obtained confirm the hypothesis that RON2 is a protein expressed in erythrocytic merozoites, as in other *Babesia* parasites.

It is known that cattle naturally infected with *Babesia* spp. in endemic areas generate antibodies that protect them from disease (Bock *et al*., 2004). To evaluate whether cattle infected naturally with *B. bigemina* generate antibodies that recognize RON2, an indirect ELISA was performed. The results showed that 98.26% of the infected cattle had antibodies that recognized peptide A, while 99.13% of the cattle had antibodies that recognized peptide B. For the difference in the sera recognition of the two different peptides we can only speculate that since B-cell epitope recognition is influenced by antigenic dominance, these two peptides contain B-cell epitopes with different immunogenicity, therefore, they do not generate the same antibodies titters in the same animals. Our finding supports the fact that RON2 is recognized by the immune system of cattle naturally exposed to *B. bigemina* in endemic areas. These results together with those described by Hidalgo-Ruiz *et al*. (2018), demonstrate that conserved B-cell epitopes of RON2 are implicated in humoral immune responses in bovine babesiosis under natural conditions. Since the cattle sera analyzed were obtained from nineteen farms in four different states in Mexico, where bovine babesiosis antigens have been reported to be highly variable (Borgonio *et al*., 2008; Genis *et al*., 2008), our findings support the hypothesis that RON2 is highly immunogenic and contains conserved B-cell epitopes, as it was previously described in *P. vivax* (Bittencourt *et al*., 2018; López *et al*., 2018). However, broader analyses including sera from other endemic countries are needed to confirm this hypothesis. Additionally, since both peptides designed in this work have a high percentage similarity with RON2 of *B. bovis* (82 and 88%, for peptide 1 and peptide 2, respectively), there is a high probability of cross-reaction, and more studies are necessary to test this hypothesis. However, while not definitive, the failure of the sera to react with *B. bovis* iRBCs by IFAT indicate that it is unlikely that the ELISA reactions were due to infection of any individual animal with *B. bovis*. Finally, to test the capacity of anti-RON2 antibodies to block invasion of erythrocytes, an *in vitro* neutralization assay was performed. A reduction in the percentage of PPE of 62.22 and 51.28% for peptide A and peptide B, respectively, confirmed this hypothesis. These results were expected, since RON2 induces invasion-blocking antibodies in other *Babesia* species. For example, Ord *et al*. (2016), demonstrated that RON2 is able to inhibit the erythrocyte invasion by *B. divergens* up to 44%. Even when we obtained similar results to those described by Hidalgo-Ruiz *et al*. (2018), it is worth noting that when *B. bovis* RON2 peptides were individually tested in an *in vitro* neutralization assay, antibodies against both peptides did have an additive effect, which was not observed in this study. Moreover, the PPE diminished when they were evaluated together. This could be due to several factors, including dilution of each antiserum in the mix (50% each), allosteric interference or different antibody titers, which were not determined in this study. More studies are needed to test these hypotheses. It has been reported for *Plasmodium yoelii* that antibodies against a peptide complex of AMA1-RON2 reached a complete inhibition (Srinivasan *et al*., 2014) by an apparent disruption of the interaction between these proteins. In our studies, *B. bigemina* AMA-1 was not evaluated; however, our findings suggest that *B. bigemina* RON2 could be considered as a part of a multiantigen vaccine.

In summary, this study demonstrates that *B. bigemina* has a *ron2* gene and that the predicted protein contains a CLAG domain, a key feature in RON2 like other Apicomplexa. In *B. bigemina*, RON2 is expressed in merozoites and contains conserved B-cell epitopes. Importantly, RON2 is recognized by naturally infected cattle and induces neutralizing antibodies. All of this is consistent with the ideal characteristics for vaccine or diagnostic candidates against bovine babesiosis caused by *B. bigemina*.

## Accession number

The sequence obtained from the *B. bigemina* Chiapas strain in this study was submitted to GenBank (National Center for Biotechnology Information, https://www.ncbi.nlm.nih.gov/nucleotide) with the accession number KU696964.
